# The role of suction thrust in the metachronal paddles of swimming invertebrates

**DOI:** 10.1038/s41598-020-74745-y

**Published:** 2020-10-20

**Authors:** Sean P. Colin, John H. Costello, Kelly R. Sutherland, Brad J. Gemmell, John O. Dabiri, Kevin T. Du Clos

**Affiliations:** 1 Roger Williams University, Bristol, RI 02809 USA; 2 Marine Biological Laboratory, Woods Hole, MA 02543 USA; 3 Providence College, Providence, RI 02918 USA; 4 University of Oregon, Eugene, OR 97403 USA; 5 University of South Florida, Tampa, FL 33620 USA; 6 California Institute of Technology, Pasadena, CA 91125 USA

**Keywords:** Biomechanics, Mechanical engineering, Behavioural ecology

## Abstract

An abundance of swimming animals have converged upon a common swimming strategy using multiple propulsors coordinated as metachronal waves. The shared kinematics suggest that even morphologically and systematically diverse animals use similar fluid dynamic relationships to generate swimming thrust. We quantified the kinematics and hydrodynamics of a diverse group of small swimming animals who use multiple propulsors, e.g. limbs or ctenes, which move with antiplectic metachronal waves to generate thrust. Here we show that even at these relatively small scales the bending movements of limbs and ctenes conform to the patterns observed for much larger swimming animals. We show that, like other swimming animals, the propulsors of these metachronal swimmers rely on generating negative pressure along their surfaces to generate forward thrust (i.e., suction thrust). Relying on negative pressure, as opposed to high pushing pressure, facilitates metachronal waves and enables these swimmers to exploit readily produced hydrodynamic structures. Understanding the role of negative pressure fields in metachronal swimmers may provide clues about the hydrodynamic traits shared by swimming and flying animals.

## Introduction

Broad surveys of propulsors of swimming and flying animals (e.g.; fins, wings, limbs) have shown that most animal propulsors bend similarly in location (inflexion point occurs ~ 0.65 along propulsor) and excursion (they bend ~ 27°)^1^. To understand the basis of these patterns we must better understand how propulsors interact with their surrounding fluid.

Drag-based paddling is a common propulsive mode used for swimming by a variety of animal phyla moving within all aquatic ecosystems. Paddlers can range from the smallest invertebrate larvae to large crustaceans and mammals. To generate thrust, propulsive paddles—e.g.; a limb, fin or ctene—initiate a power stroke and the drag acting on the paddle generates thrust in the opposite direction of the paddle’s motion. Animals are propelled forward as long as the drag acting on the paddle during the power stroke is sufficient to overcome the resisting drag on the animal’s body and the drag generated by the paddle on its return during the recovery stroke^2^. To maximize thrust, the shape and kinematics of the propulsor must be controlled so that the propulsor maximizes the drag it generates during the power stroke and minimizes its drag during the recovery. While this may appear fundamentally simple, the hydrodynamics of this process can be quite complex and may account for the observed diversity in the shape and kinematics of paddles among swimming animals.

The hydrodynamics around a moving propulsor generate a pressure field along the propulsor surface. The pressure gradient across the propulsor is the ultimate source of thrust necessary for swimming (and flying). Conventionally, it has been thought that these pressure fields are dominated by positive pressure generated as a propulsor pushes on the water and pushes the swimmer forward^3^. However, recent studies have shown that for some animals, including fish and jellyfish, it is actually negative pressure fields aligned along the leeward side of moving propulsors that dominates the pressure field and serve to essentially pull animals through the water (termed suction thrust^4^). However, capacities to non-invasively quantify pressure^5^ and forces across a propulsor^4,6^ are recent innovations and the prevalence of suction thrust among different propulsive strategies has not been well investigated. In particular, the role of suction thrust in drag-based paddling has not been examined.

**Figure 1:**
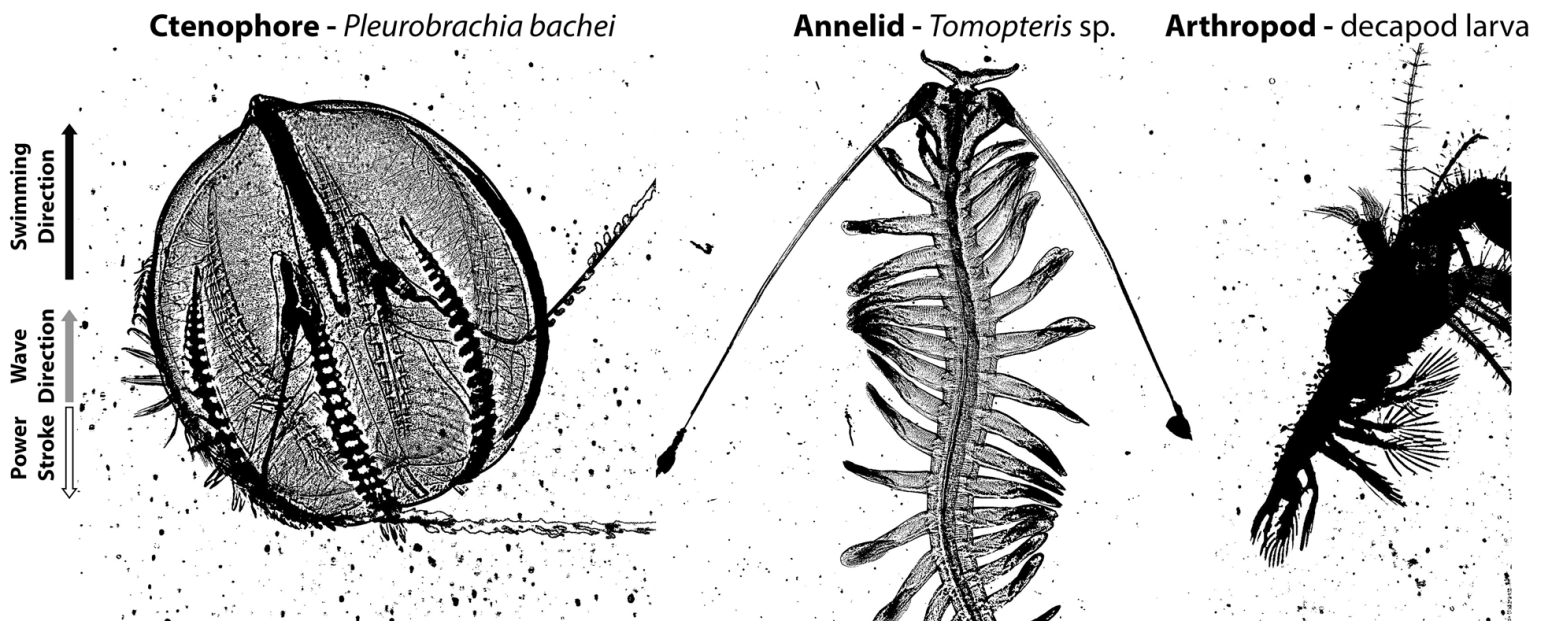
Brightfield images of three taxonomically diverse marine invertebrates, the ctenophore *Pleurobrachia bachei*, the polychaete annelid *Tomopteris* sp. and an unidentified larval decapod arthropod, left to right. Each of these swim using multiple paddles that move sequentially with antiplectic metachronal wave kinematics. As such, the sequential wave of paddles passes in the opposite direction of the power stroke.

Understanding drag-based paddling is further complicated for animals with multiple propulsor units. These designs are very common among swimming arthropods—which have sets of swimming legs—and polychaete worms—which have many parapodia—and ctenophores—which have rows of fused cilia called ctenes (Fig. 1). While there is large diversity in the morphology of these propulsors, the coordination of multi-unit propulsors appears to be highly conserved as antiplectic metachronal waves among a diverse array of animals. Antiplectic waves are characterized by a power stroke direction that is opposite in direction from the metachronal wave itself (Fig. 1). This kinematic coordination of multiple propulsors has been proposed to be widespread because antiplectic metachronal waves enable each propulsor to move unobstructed by adjacent propulsors^7^. Despite the kinematic benefits, the complex spatial and temporal aspects of the hydrodynamics near the propulsors have made quantifying the flow difficult^8^. Consequently, few studies have quantified the flow around metachronal propulsors^8,9^ and most studies on thrust generation by metachronal swimmers have focused on trailing wakes produced by metachronally-coordinated propulsors. While the focus on wake forms can provide estimates of whole-body thrust, it cannot provide information on the mechanism generating thrust as the propulsor surface. It is this fluid–structure interaction at the propulsor scale that ultimately determines the wake forms that are more commonly measured.

In order to identify unifying characteristics of multi-unit propulsors that may account for conserved kinematics observed between animal phyla, we quantified the hydrodynamics of a diverse array of evolutionarily distinct aquatic swimmers from three different phyla (Ctenophora, Annelida, Arthropoda) with multi-unit propulsors (Supplementary Table S1). To achieve this, our goal was to use high-resolution micro-particle image velocimetry (μPIV)^10^ to quantify the fluid motions immediately adjacent to propulsive limbs of these multi-unit swimmers at the scale of the limbs. All of these animals swim using drag-based paddles with antiplectic metachronal kinematics and at Reynolds numbers (based on appendage length) ranging from 17 to 95 (Supplementary Table 1). To quantify the hydrodynamics around their propulsors, experiments were conducted in transparent laboratory vessels containing natural seawater using μPIV^10^. This non-invasive approach relies on a high-speed camera with a narrow focal plane (< 10 μm) to simultaneously image the motion of the propulsors and particles (< 5 μm) immediately adjacent to the moving propulsors during the power stroke of the propulsors. The velocity fields measured using PIV software (DaVis by LaVision, Inc.) were subsequently input to custom algorithms to compute corresponding pressure fields and forces surrounding the propulsors; see “Methods” for further details.

## Results

**Figure 2:**
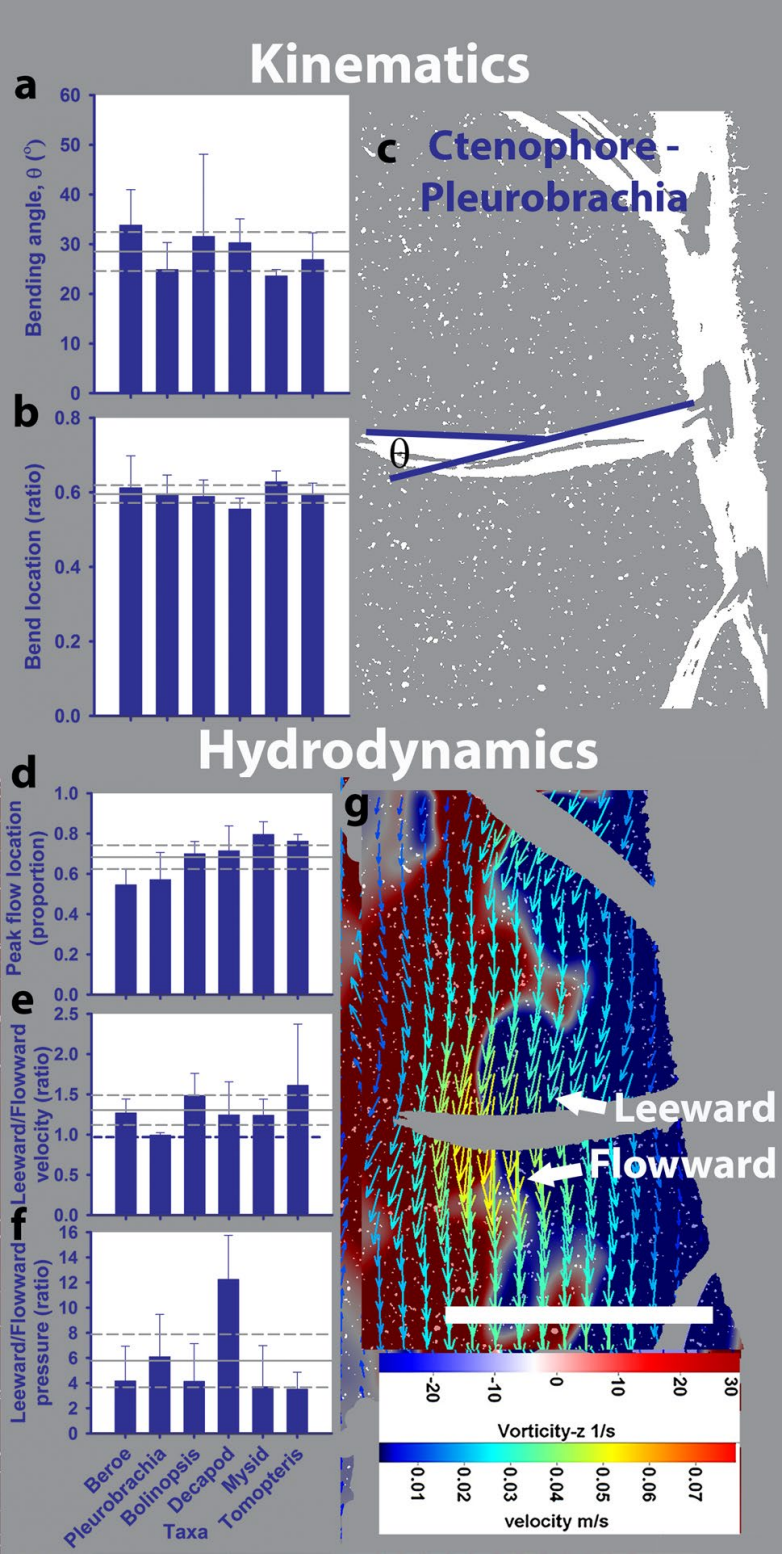
Kinematics and hydrodynamics around paddles of six morphologically and taxonomically diverse species. (**c**) and (**g**) are representative images showing the kinematic (**c**) and hydrodynamic (**g**) measurements of the ctenes of ctenophore *Pleurobrachia brachei* swimming up. The ctenes and limbs of the five species analyzed bent similarly during their power stroke, whereby, (**a**) the mean bending angle among all the species was 27° ± 6.1 (mean shown by solid line and s.t. dev. shown by dashed line) and (**b**) the mean inflexion point among all the species was located 0.59 ± 0.045 along the the limb/ctene. The (**d**) peak water flow (i.e., maximum flow velocity) was located near the inflexion point occurring on average at 0.68 ± 0.1 along the limb/ctene. In addition, (**e**) water flow was on average 1.31 ± 0.22 times greater on the leeward side of the limb/ctene than the flowward side (blue dashed line shows unity). The (**f**) average negative pressure along the length of the leeward side of the limb/ctene was 5.65 ± 3.3 times greater than the average positive pressure along the flowward side. Error bars represent the s.t. dev among replicated individuals of each species (n = 3). The white scale bar in (**g**) is 1 cm in length.

The propulsors—i.e., ctenes and limbs—of all the animals examined move sequentially during their power stroke as an antiplectic metachronal wave (Fig. 1). In addition to sharing wave kinematics, the propulsors of all the species examined bent similarly during the power stroke, in that they did not differ in how much they bent (Fig. 2a,c; bending angle = 27° ± 6.1; ANOVA, df = 5, 12, f_stat_ = 0.71, p = 0.6) or where they bent along the propulsor (Fig. 2b,c; percent distance along propulsor = 59.2% ± 4.5; ANOVA, df = 5, 12, f_stat_ = 0.73, p = 0.6). The material composition and structural organization of the different propulsors varied between phyla and we do not know whether the bending at propulsor tips was actively controlled or resulted from a passive response to movement of the propulsor base. Among ctenophores, ctene bending appears to be passive (as there are not muscle cells in the ctenes) and they have the highest variability observed among the taxa (Fig. 2a). The example image of the *Pleurobrachia* ctene bending is at 18.5° (which was on the lower end of the bending angles observed among the measured individuals) and it bent at a location 55.1% along the propulsor (Fig. 2c).

**Figure 3:**
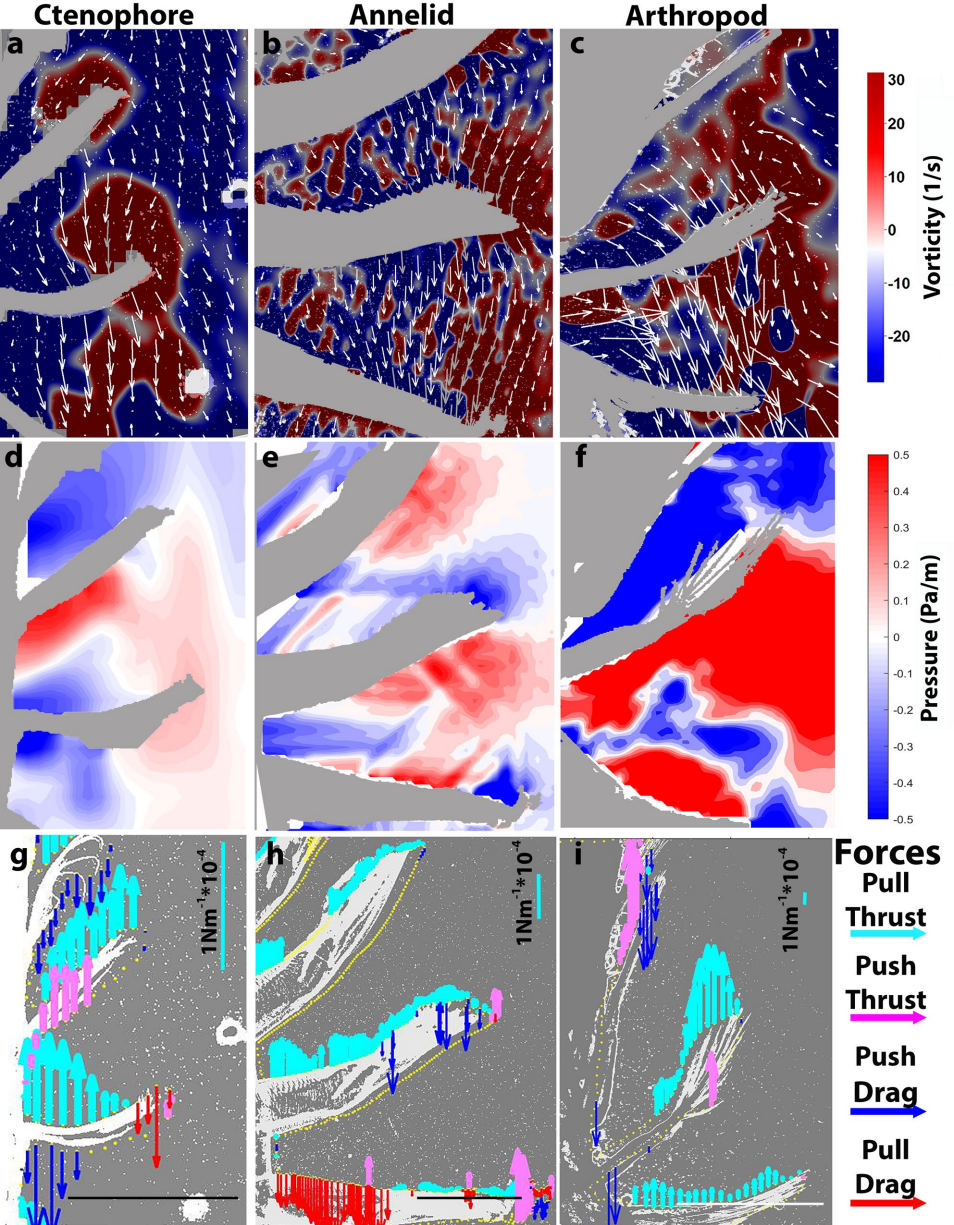
Representative PIV, pressure and force vectors of (**a**,**d**,**g**) the ctenophore *Pleurobrachia bachei*, (**b**,**e**,**h**) the polychaete annelid *Tomopteris* sp. and (**c**,**f**,**i**) an unidentified larval decapod arthropod swimming up. The deep blue areas in the pressure frames (**d**–**f**) indicate areas of strong negative pressure. The aquamarine force vectors show the predominance of the pulling forces acting on the limbs/ctenes during the power stroke. Scale bars along the bottoms show 1 mm length.

Similarly to results for other bending propulsors^11,12^, the vorticity fields adjacent to each moving propulsor aligned around the bend of the propulsive limb or ctene. Velocities of the fluid adjacent to the propulsors varied along the length of the propulsor (Figs. 2g, 3a–c; moving from the base to the tip of the propulsor) and peaked in the proximity of the bending inflexion point rather than at the very tip of the propulsor (Fig. 2d; peak flow location = 68.4% ± 10.0). Interestingly, velocities on the leeward side of the propulsors were greater than on the flowward side (Holm-Sidak non-parametric test, P < 0.05; Fig. 2e). These velocity fields are different than velocities around rigid paddles where peak velocities occur at the very tip and on the flowward side of the propulsors^13,14^.

Pressure fields around the bending propulsors (Fig. 3d–f) revealed that these velocity characteristics were related to strong negative pressure regions along the leeward side of the propulsors (Figs. 2f, 3d–f). Weaker positive pressure regions were observed on the flowward sides of the propulsors. Throughout the propulsors’ power stroke, negative pressure was much greater than positive pressure (Fig. 2f), which meant the limbs and ctenes pulled a bulk of fluid toward the rearward direction relative to the animal’s trajectory.

**Figure 4:**
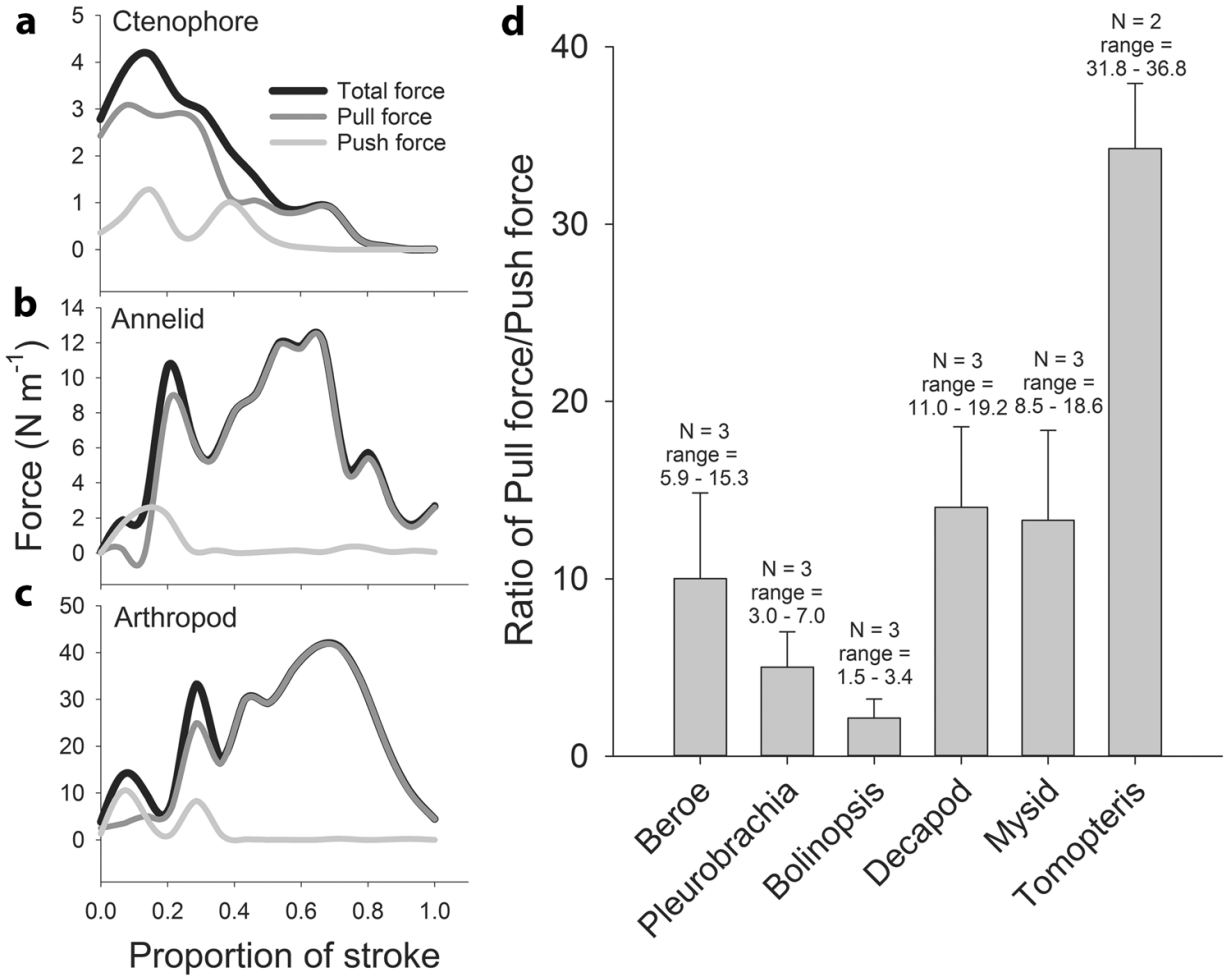
Representative time series of pull (−), push (+) and total forces summed around the limb/ctene of (**a**) the ctenophore *Pleurobrachia bachei*, (**b**) the polychaete annelid *Tomopteris* sp. and (**c**) an unidentified larval decapod arthropod. All the time series show that push (+) forces are initially high at the beginning of the power stroke but then pull (−) forces dominate the total force acting on the limb/ctene for the rest of the power stroke. (**d**) The ratio of the pull/push forces (averaged over the power stroke) for a range of ctenophores (*Beroe*, *Pleurobrachia*, *Bolinopsis*), arthropods (unidentified decapod larvae and mysid shrimp) and annelid (*Tomopteris*) show that for all these diverse paddlers that use metachronal paddles, negative pull forces dominate the forces generating thrust during their power stroke.

Pressure values along the surface of the propulsors can be used to estimate the thrust and drag acting on the propulsors throughout their power strokes (Fig. 3g–i). The negative pressures on the leeward side of the propulsors resulted in all the propulsors predominately pulling the animals forward rather than pushing the animals forward (Fig. 4a–d). Time series of the forces during the power stroke showed that the pushing forces initially, and briefly, peaked as the propulsors began their stroke, but, for the majority of the power stroke the propulsors were primarily pulling the animals forward (Fig. 4a–c). As a result, the sum of the forces acting on the propulsor throughout the power stroke showed that the pulling forces strongly dominated the forces generated by the metachronal propulsors. Among all the ctenophores, arthropods and the annelid examined, the pulling forces were 2 to 34 times greater than the pushing forces generated by the metachronal propulsors (Fig. 4d).

## Discussion

The goal of this study was to examine the fluid flows directly adjacent to propulsor surfaces in order to better understand how metachronal propulsors interact with fluids for thrust production. Based on the direct comparison of the mean contributions of pulling vs. pushing forces throughout the power stroke of replicate individual propulsors (Fig. 4, which are generated by negative vs. positive pressure fields, respectively) we suggest that the propulsors of the animals examined rely predominantly on negative pressure for generating thrust. The assertion that these propulsor level observations to apply to the movement of the whole animal requires the assumptions that, first, the propulsors we quantified are representative of the all the other propulsors contributing to swimming thrust, and second, that the thrust generated for the whole animal is due to accumulated total thrust generated by individual propulsors. Although our data addresses the first of these assumptions by replicating individual propulsors, we cannot document the second assumption that the total thrust represents the sum of all individual propulsor elements. While this second assumption is intuitively appealing, our data is confined to the small spatial and temporal scales around individual propulsor elements. Confirmation that the whole-organism thrust results from of the summation of individual contributions requires experiments at different scales than those used in the current study.

Thrust generated by a propulsor is ultimately determined by the overall pressure gradient across the propulsor. So does it matter whether that gradient is dominated by negative or positive pressure? We believe that this distinction is fundamental for understanding why animal propulsors bend in a surprisingly characteristic and narrow range. Rigid paddle designs are dominated by positive pressure pushing against a fluid, which in turn, generates thrust pushing a body forward. Bending at propulsor margins encourages vortex formation on the lee side of the propulsor (Figs. 2, 3) that differs from rigid propulsors. Counter-rotating vortices formed on the lee side of a bending propulsor accelerate fluid at the intersection of the vortices^12,15^. The fluid thus accelerated relative to the leading edge of the propulsor is the basis of the pressure gradient across the propulsor surface. In turn, this elevated pressure gradient generates high thrust and is the reason for the dominant contribution of suction thrust to natural bending propulsors. More generally, negative pressure fields are a fundamental feature of vortices which are universally formed around objects moving in fluids (except at the lowest Reynolds numbers). Lift, a different propulsive mode that relies on negative pressure, is a well-known example that illustrates how kinematics and morphology can enhance negative pressure for thrust^2^. Lift occurs when a foil separates flow traveling over and under the foil surface. With the correct foil shape and kinematics, the separation of flow can generate strong negative pressure fields above the foil leading to an upward pulling thrust on the foil. This lift relies on the negative pressure field and foil shape.

To be clear, the thrust generated by limbs and ctenes in this study is not lift because the forces generated by lift are directed perpendicular to the direction of flow and the forces we describe are oriented in the direction of flow (Fig. 3). However, like lift, we suggest that paddles must move with prescribed kinematics to generate enhanced negative pressure fields. Bending kinematics in particular have been shown to greatly enhance vorticity and along with that, negative pressure^11,16,17^. Rigid, non-bending paddles generate different hydrodynamic structures than we observed^13,14^ and do not generate strong negative pressure fields^16–19^. Therefore, the kinematics of bending appear to be important for generating strong negative pressure fields around moving propulsors.

Until recently, technical constraints have limited our ability to investigate the scope of the benefits of using negative pressure for thrust. However several numerical studies, and a few experimental studies, have compared rigid to flexible propulsors. These studies have demonstrated that first, bending enhances negative pressure fields, second, bending generates elevated thrust, and third, bending enhances hydrodynamic efficiency^12,16,17,20–24^. The hydrodynamic patterns around bending propulsors show that negative pressure fields associated with bends generate significantly greater flow velocities than positive pressure fields (Fig. 2e^11,12,16,21^). This would lead to enhanced momentum transfer and explain the enhanced thrust observed for bending propulsors. The similar bending kinematics between the limbs and ctenes in this study and the swimming and flying animals from Lucas et al.^1^ suggests that these small paddling swimmers may employ similar hydrodynamic features as flying birds and swimming fish. If these bending patterns are predominately used to generate negative pressure fields for thrust, it follows that there is a need for greater focus on negative pressure around bending propulsors in order to understand the extent of the benefits of animals experience by pulling rather than pushing themselves through fluids.

Despite the vast difference in scale and Reynolds number, the results of this study suggest that the small metachronal paddles of swimming invertebrates may produce some similar effects as flapping wings in birds and insects. For example, there are similarities in the degree of bending and location of bending for the paddles in this study and the spanwise flexibility of birds and insects^1^. Such spanwise flexibility was found to be beneficial and yielded an increase in thrust coefficient, and a small decrease in power-input requirement, resulting in higher efficiency^25^.

In addition to the benefits for single propulsors, negative pressure fields can facilitate the movement and coordination of multiple propulsors which have antiplectic metachronal wave kinematics. During an antiplectic metachronal wave, a leading propulsor will begin the power stroke and, after it has initiated its stroke, the propulsor immediately behind it will initiate its own power stroke. This sequential pattern will continue for all the subsequent propulsors in the antiplectic wave. The predominately negative pressure on the leeward of each propulsor can serve to facilitate the kinematics of the adjacent propulsor by reducing the hydrodynamic resistance necessary to initiate and complete its power stroke^26^. In addition, the negative pressure in the gap between adjacent propulsors can serve as a cue for the adjacent propulsor to initiate its power stroke. It has been suggested that the ctenes of ctenophores require such cues to coordinate the metachronal kinematics^26–28^. At lower Reynolds numbers (Re < 10^–2^ versus the 10^1^ we observed), adjacent cilia have been shown to be hydrodynamically synchronized^29^. Although the role of negative pressure at these Re have not been investigated, negative pressure may serve as the hydrodynamic mechanism that coordinates these propulsors as well as those at higher Re levels. It may be possible that antiplectic metachronal waves inherently depend upon negative pressure and that the predominance of antiplectic waves in multi-unit propulsors may result from the predominance of negative pressure fields that assist coordination of multi-unit propulsors.

The limbs and ctenes of small invertebrates rely on the generation of negative pressure fields for thrust production and their bending kinematics conform to the bending patterns observed among a vast array of swimming and flying animals. This may reflect the fact that a subtle bend (< 30% inflexion) appears to generate a cascade of hydrodynamic effects which enhance formation of negative pressure regions around propulsors and, ultimately, lead to enhanced thrust and hydrodynamic efficiency. These enhancements appear to be optimized for small bends^20,21^. Invertebrate animals with multiple paddles can coordinate the sequential build-up of negative pressure fields along antiplectic metachronally moving limbs for an additive thrust benefit and to facilitate the coordination of the sequential propulsors.

## Detailed methods

All of the metachronal swimming animals used in this study (ctenophores: *Pleurobrachia bachei*, *Bolinopsis infundibulum*, *Beroe* sp.; crustaceans: unidentified decapod larva, unidentified mysid shrimp; polychaete: *Tomopteris* sp.; Supplementary Table S1) were hand-collected from docks at Friday Harbor Labs in May and June of 2014, 2016 and 2017. Individuals were maintained in running seawater tables at ambient environmental temperatures (10–12 °C). Prior to filming individuals were gently transferred to glass vessels and observations were made within 24 h. of collection. Glass vessels ranged in size from 4 × 1 × 2 to 15 × 4 × 12 (height × depth × width in cm) to accommodate animals of varying sizes.

### Kinematics

For kinematic measurements, individuals were illuminated using a brightfield collimated light set-up with a 10× long-working-distance objective^10^. Images were collected with high speed monochrome video cameras at 500–6400 frames per second at a resolution of 2048 × 2048 pixels. Image stacks were imported into Fiji ImageJ software to quantify the swimming and limb/ctene kinematics. For larger field of view images of whole animals, the same brightfield set-up was used with a 100 mm Nikon lens. Since the focus of this study is to understand how the metachronal propulsors generate thrust we measured the kinematic properties during the mid-power stroke.

### Fluid mechanics

To examine the relationship between kinematics and fluid mechanics, we used brightfield imaging to obtain shadowgraph micro particle image velocimetry (μPIV)^10^ on free swimming animals (see Supplementary Table 1 for velocities). The brightfield PIV set up was identical to the set up for kinematics but the water was seeded with *Isochrysis galbana* cells (approx. diameter = 5 µm). Image sequences during swimming were selected where the limb or ctene was in the focal plane throughout the stroke cycle. The focal depth for the 10× objective is very narrow (approx. 8 µm) and few sequences satisfied this criteria which greatly limited the number of replicate sequences of free swimming animals. Image pairs were subsequently analyzed in DaVis 8.3.1 (LaVision GmbH, Goettingen, GER) using a cross-correlation PIV algorithm with a decreasing interrogation window size of 64 × 64 pixels to 32 × 32 pixels or 32 × 32 pixels to 16 × 16 pixels with 50% overlap to produce velocity vectors and vorticity contours.

### Pressure and torque measurement

Velocity fields collected via PIV were input to a custom program in MATLAB that computed the corresponding pressure fields. The algorithm integrates the Navier–Stokes equations along eight paths emanating from each point in the field of view and terminating at the boundaries of the field of view. As the full 2D Navier–Stokes equations are used, the pressure calculations include the full viscous effects present in the Reynolds number range of the ctenes. The pressure at each point is determined by computing the median pressure from the eight integration results. Limbs and ctenes were masked prior to computation to prevent surface artefacts in the pressure and torque results. Masks were generated using a custom MATLAB (MathWorks, Inc.) program that automatically identified the boundary of the animal body based on image contrast at the interface between the animal body and the surrounding fluid, and body outlines were smoothed prior to later analyses. These methods have been previously validated against experimental and computational data, including numerical simulations of anguilliform swimming^5^ and direct force and torque measurements of a flapping foil^6^. The MATLAB code is available for free download at https://dabirilab.com/software.

The fluid force normal to the body surface due to the local fluid pressure was determined by integrating the calculated pressure along the corresponding surfaces of the body^6^. Validations against measurements made on physical models show that these calculation techniques based on 2D PIV images are robust to a small degree of out-of-plane flow such as that induced by a fish’s slight rolling motions during turns, so long as the fish remains centered in the imaging plane^6^. The body outline of each animal was divided into segments of equal length for spatial integration. Because the surface geometry was specified in a single plane, the force calculations were evaluated per unit depth (i.e. giving units of Newtons per meter of depth perpendicular to the measurement plane.

## Supplementary information

Supplementary Information.
