# Supplementary material for: The role of suction thrust in the metachronal paddles of swimming invertebrates
Source: Sci Rep. 2020 Oct 20;10:17790. doi: 10.1038/s41598-020-74745-y (PMC7576154; doi:10.1038/s41598-020-74745-y)
Supplement: Supplementary file 1 — Supplementary Information. [file 41598_2020_74745_MOESM1_ESM.docx]

**The role of suction thrust in the metachronal paddles of swimming invertebrates**

Colin, Sean P.^1,2^*, Costello, John H.^2,3^, Sutherland, Kelly R.^4^, Gemmell, Brad J.^5^, Dabiri, John O.^6^, DuClos, Kevin^5^

1. Roger Williams University, Bristol, RI 02809,USA, [scolin@rwu.edu](mailto:scolin@rwu.edu)
2. Marine Biological Laboratory, Woods Hole, MA 02543, USA
3. Providence College, Providence, RI 02918, [costello@providence.edu](mailto:costello@providence.edu)
4. University of Oregon, Eugene, OR 97403, [ksuth@uoregon.edu](mailto:ksuth@uoregon.edu)
5. University of South Florida, Tampa, FL 33620, [bgemmell@usf.edu](mailto:bgemmell@usf.edu), kduclos@usf.edu
6. California Institute of Technology, Pasadena, CA 91125, [jodabiri@caltech.edu](mailto:jodabiri@caltech.edu)

**Supplementary Information**

Supplementary Table S1. The morphological, kinematic and Reynolds number data of the animals used in the study (n = 3).
